# Identifying disparities in patient-centered care experiences between non-Latino white and black men: results from the 2008-2016 Medical Expenditure Panel Survey

**DOI:** 10.1186/s12913-020-05357-5

**Published:** 2020-06-03

**Authors:** Jamie A. Mitchell, Ed-Dee G. Williams, Yuyi Li, Wassim Tarraf

**Affiliations:** 1 School of Social Work, University of Michigan, Ann Arbor, MI USA; 2 Department of Sociology, University of Michigan, Ann Arbor, MI USA; 3 Department of Electrical and Computer Engineering, University of Detroit Mercy, Detroit, MI USA; 4 Institute of Gerontology, Wayne State University, Detroit, MI USA; 5 Department of Healthcare Sciences, Wayne State University, Detroit, MI USA

**Keywords:** Patient-centered, Minority, Gender, Primary care

## Abstract

**Background:**

Patient-centered healthcare in the context of a medical home (PCMH) is an important pathway to reducing healthcare inequities. To date, no work has examined the prevalence of care experiences associated with PCMH among non-elderly Black males.

**Methods:**

We analyzed data, on 22 indicators representative of six healthcare domains associated with PCMH experiences, from non-Latino White (NLW) and Black males aged 18–64 from the 2008–2016 Medical Expenditure Panel Survey (*n* = 47,405). We used generalized linear models to test whether *Behavioral Model* factors attenuate any differences in access to these domains between NLW and Black males, and decomposition techniques to examine the contribution of these factors to reported differences.

**Results:**

Black males reported 1) lower access to personal primary care providers, 2) poorer quality communication with providers, and 3) lower levels of care comprehensiveness (all *p* < 0.05). Differences between groups were attenuated but not eliminated by accounting for the *Behavioral Model* factors particularly through enabling and predisposing factors. Group health characteristics were not a primary driver of racial differences in care experiences across all the considered domains.

**Conclusions:**

Black men, in the U.S, continue to face barriers to accessing high quality, patient-centered care, specifically as it relates to accessing specialty care, medical tests, and patient-provider communication.

## Background

Black Americans experience persistent disparities in health outcomes, and disproportionately lower quality healthcare. Patient-centered healthcare models have shown promise in reducing the occurrence of disparities among medically underserved groups, including Black Americans. A report by the Institute of Medicine [1] describes patient-centered care (PCC) as a gold-standard measurement of quality care for all patients that encompasses improved training for physicians, clinicians and other professionals across diverse health fields. PCC positions the patient as the priority and makes health care services and care adaptive to the patient rather than requiring the patient to adapt to the care [1] PCC employs more holistic healthcare standards that incorporate the values, beliefs and specific needs of patients and their families [2, 3]. By focusing on the patient rather than the disease or illness only, PCC is a concept that embodies four common core beliefs: holistic, individualized, respectful, and empowering [4] . Research on PCC has focused on the philosophy of care as well as the implementation across healthcare sectors.

### Black Americans and PCC

The interaction between patients and their healthcare providers, namely physicians, is a powerful indicator of patient outcomes, including satisfaction, adherence to treatment recommendations, and likelihood of returning for follow-up care [2, 4, 5]. Black Americans rate interpersonal care received by physicians more negatively than their White counterparts [6] and studies have found that Blacks receive poorer interpersonal communication via lower levels of “rapport building and use of affective tone”, heightened levels of physician verbal dominance, less patient-centeredness, and shorter medical visits [7, 8]. Furthermore, Blacks and other racial/ethnic minorities are less likely to receive referrals for medical specialists, to have a consistent usual source of care, to have comprehensive health insurance or to trust the healthcare system; and they experience greater difficulties in navigating the healthcare system [9, 10]. Studies measuring Black patients’ trust of physicians also find that high levels of mistrust are associated with less medication adherence and physician communication [11]. However, research on how Black men in particular, are served by patient-centered models of care, given the burden of health disparities they face, is insufficient. For example, Black men have one of the shortest life expectancies in the U.S and disproportionately high prevalence rates for several life-threatening or disabling diseases such as kidney failure, heart disease, cancer and hypertension [12, 13] when compared to their White counterparts. These disparate outcomes have been partially linked to individual behaviors amenable to change, and factors such as low socio-economic status, masculine gender norms that discourage medical help seeking, and limited health literacy [14, 15]. However, poor health care access and quality remain critical to perpetuating health disparities for this population, and improved health communication and longer medical visits are associated with higher trust in physicians and relatedly, greater reported quality of healthcare [7].

### PCC and patient-centered medical homes

Patient-Centered Medical Homes (PCMH) arose in response to the need to implement patient centeredness within a constant care setting [16]. Patient-centered medical homes physically co-locate healthcare services and reduce transportation barriers that might normally make it difficult for patients to access care across multiple outpatient locations [3, 16]. PCMH make use of team- based medical services whereby the patient’s primary care physician leads a team of other health care providers focused on their specific needs; care is coordinated throughout the healthcare system and community, consequently improving access to different aspects of care. Ideally, patients receive same day appointments and more direct access to physicians and specialists [17, 18] . This model looks to improve healthcare access and quality while minimizing healthcare disparities. The current study considers how healthcare indicators of a PCMH model is experienced by Black men in relation to non-Latino White (NLW) men in a nationally representative data set.

## Methods

### Sample

We used data from the 2008–2016 Medical Expenditures Panel Survey (MEPS). The MEPS is a comprehensive survey on health services use and expenditures for the non-institutionalized, civilian United States population. In addition to the collected data on use and expenditures, the MEPS include detailed information on participant demographics, insurance, and health status and conditions. The MEPS analytic subpopulation utilized for the current study were eligible adult males 18–64 years of age who self-reported their race as non-Latino White or Black (*n* = 47,799). We excluded from our analyses participants with missing data on any of our model covariates (*n* = 394) for a final unweighted analytic sample of *n* = 47,405; the average weighted yearly equivalent of 59.7 and 11.1 million adult NLW and Black males, respectively. The MEPS has a complex survey design including stratification, clustering, and probability weighting. All analyses accounted for the complex design of the MEPS to ensure appropriate generalization and inferences to the intended target population. Specifically, we used a Taylor Series Linearization approach to variance estimation as implemented through the survey functionalities in the Stata v.15 software. All data are publicly available from the Agency for Healthcare Research and Quality (AHRQ). Study protocol qualified for “Exemption” according to the Department of Health and Human Services Code of Federal Regulations.

### Outcomes

MEPS participants were surveyed in detail about their healthcare experiences. We focused on a set of questions that relate to 6 domains covering the availability of: 1) a personal primary care provider; 2) enhanced access to care; 3) quality patient-provider communication 4) patient-centered care; 5) coordination by usual source of care provider; and 6) comprehensive care. Each domain was assessed using multiple questions. For example, to investigate enhanced access to care we examined four questions that probed whether 1) the participant had easy phone access to provider during regular business hours; 2) the provider had office hours on nights or weekends; and 3) how easy it was to contact the provider during off hours if needed. Our approach to approximating domains of PCMH care have been used previously in MEPS data [19–23]. In line with prior work, all questions, when not dichotomous, were dichotomized to group “never” and “sometimes” responses into one category and “usually” and always as a second category. With the exception of the usual source of care provider indicator, all considered outcomes were modeled conditional on reporting a usual source of care. A detailed list of the questions, skip patterns, and the original scaling, used to assess each of the six domains listed above is provided in Additional file 1: Figure S1.

### Primary exposure

Self-reported racial grouping was the primary predictor of interest. Our focus was on comparing male participants reporting their race as White and ethnicity as non-Latino ethnicity to those reporting their racial background as Black.

### Covariates

We followed a modified behavioral model approach [24, 25] to model care experiences as a function of: 1) predisposing factors: race (our primary outcome), age (in years), marital status (0 = not currently married; 1 = married), and a 4-category education indicator (1 = Less than high school (HS), 2 = HS or equivalent, 3 = Some college, and 4) College or more) 2) enabling factors: a 5-category poverty status, based on Census classification as coded by the AHRQ, indicator (1 = Poor, 2 = Near-Poor, 3 = Low-Income, 4 = Middle-Income, 5 = High-Income), and insurance (1 = Private, 2 = Public, 3 = Uninsured); and 3) need factors: which included two measures of self-reported physical and mental health each measured as a 5-cateogry indicator (1 = Excellent, 2 = Very good, 3 = Good, 4 = Fair, and 5 = Poor). We also controlled for two additional variables that confound access: region of residence and survey year to account for yearly fluctuations in factors that are not directly accounted for by our model.

### Analytic plan

**Fig. 1:**
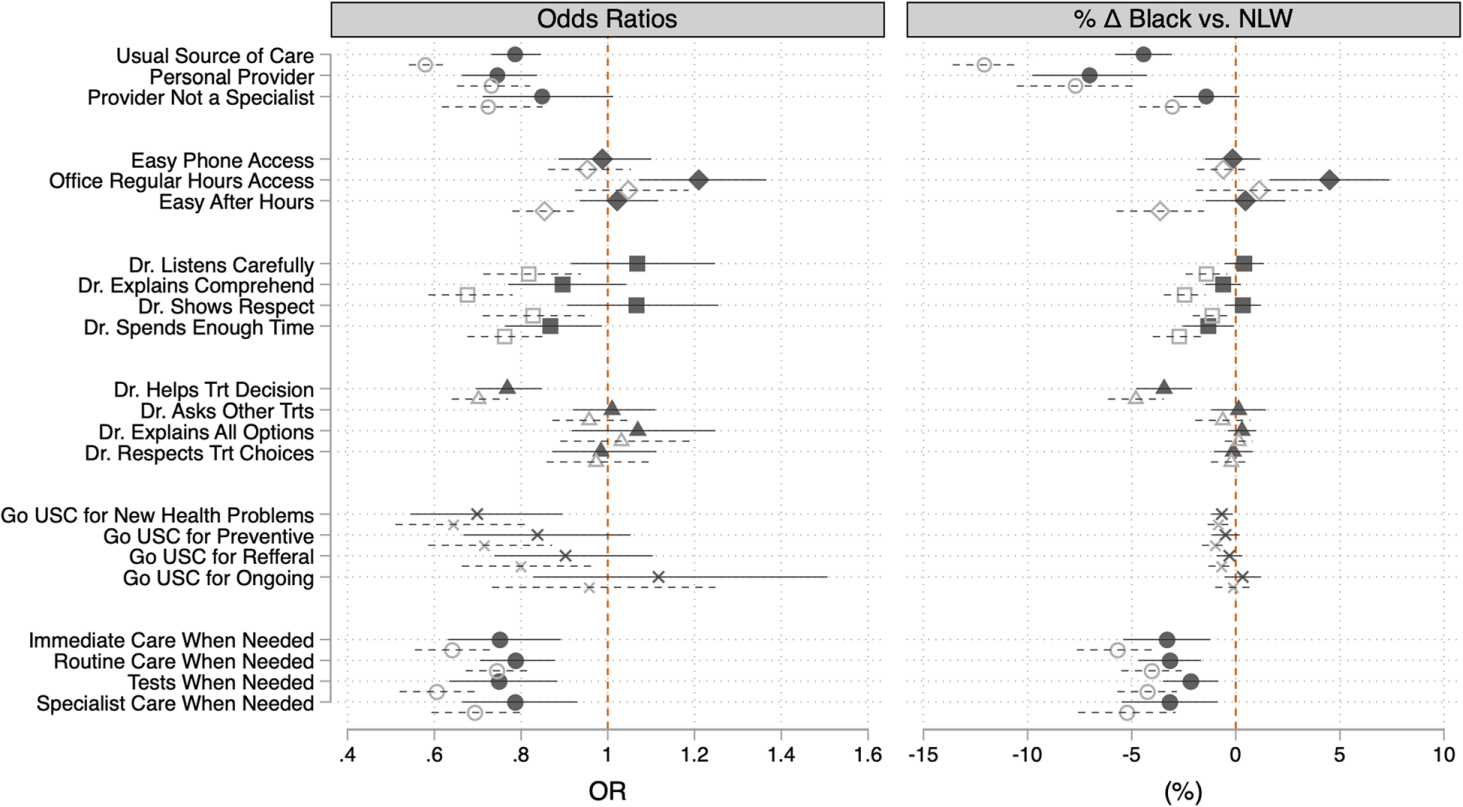
Odds Ratios (**a**), and differences in expected percentages (**b**) and 95% Confidence Intervals. Note 1: The grey hollow markers represent crude estimates. The dark markers represent estimates derived from models adjusted for the Behavioral model factors. Note 2: Values below 1 for the Odds Ratios and below 0 for the percent differences are indicative of Non-Latino White advantage on represented outcomes. Note 3: Results are derived from Logistic Regression Models using Non-Latino Whites and Black participants ages 18–64 years from the 2008–2016 Medical Expenditure Panel Survey

**Fig. 2:**
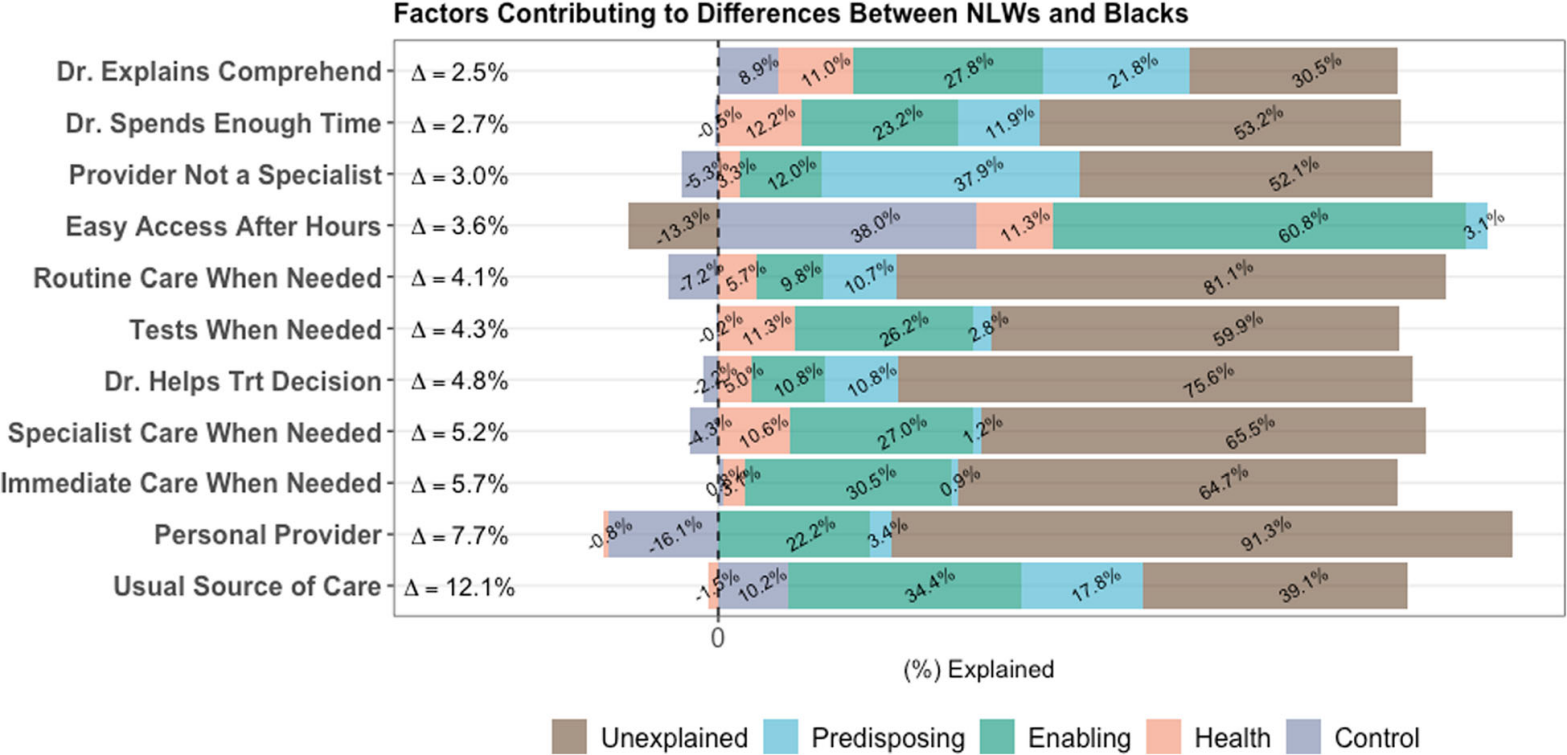
Decomposition of differences in expected probability of PCMH indicators. Note 1: The Δ in the graph represent the crude absolute difference in probability between Non-Latino Whites and Blacks. Note 2: The percentage values in the bars represent explained between group differences in the probability between Blacks and Non-Latino Whites resulting from the predisposing, enabling and health need factors; i.e. the reduction in the in difference if both groups had similar distributions on these characteristics. The unexplained bars represent the portion of crude differences that is not explained by the factors accounted for in the model. Note 3: Bars with negative values represent factors that are advantageous for Blacks. Bars with positive values represent factors that are disadvantageous for Blacks. Note 4: Results are derived from Oaxaca decomposition techniques adapted for binary outcomes using Non-Latino Whites and Black participants ages 18–64 years from the 2008–2016 Medical Expenditures Panel Survey. Note 5: Only indicators with differences between Non-Latino Whites and Blacks are statistically significant and with Δ ≥ 2% are displayed in graph

Our analyses proceeded in four steps. First, we provide descriptive statistics to characterize our sample overall and by racial group by the covariates of interest (Table 1). Second, we model the binary outcomes for each domain using survey logistic regression. For each outcome we follow a 3-step process beginning with crude (bivariate) associations (outcome as function of racial grouping), and incrementally adjusting for 1) age and 2) full set of covariates. We present a summary of the estimated models to describe differences between Blacks and NLWs in Table 2. We plot the odds ratios and 95% confidence intervals resulting from these models in Fig. 1a and provide a detailed representation of the estimated models in the Additional file 1: Tables S1–S6. Third, to facilitate the interpretation of our estimates we calculate and plot contrasts of the estimated average marginal probabilities resulting from these incremental adjustments for each outcome in each domain and their estimated 95% confidence intervals in (Fig. 1b). Fourth, to implement the institute of medicine definition of disparity, we use modified Oaxaca techniques for binary outcomes to decompose the differences in outcomes prevalence into their explained and unexplained portions. By using decomposition techniques, we can quantify the degree to which distributional differences in sets of covariates (e.g. enabling factors) across the two race groups of interest contribute to the observed (explained) differences in the prevalence of each healthcare experience outcome considered. Accessible detailed discussions of the Blinder-Oaxaca technique and its binary outcome modification have been published elsewhere [26] . We graph the decompositions for each outcome where significant differences between Blacks and NLWs are detected in Fig. 2 and present a summary of the differences and estimated explained and unexplained differences in Table 3. Detailed presentations of these decompositions are included in Additional file 1: Table S7.

**Table 1:** Demographic Characteristics of non-Latino White and Black participants ages 18–64 years from the 2008–2016 Medical Expenditures Panel Survey (Unweighted *n* = 47,405)

Age (in Years)^**a**^ (Mean)	White	Black	Overall
	42.0	39.3	41.6
**Married**^**a**^**(%)**			
Yes	56.6	35.7	53.4
**Education**^**a**^**(%)**			
Less than HS	8.6	15.3	9.6
HS or Equivalent	31.1	39.4	32.4
Some College	28.0	29.4	28.2
College or More	32.4	15.9	29.8
**Poverty**^**a**^**(%)**			
Poor	8.0	19.0	9.7
Near Poor	2.6	5.0	3.0
Low Income	9.6	15.1	10.5
Middle Income	29.7	32.3	30.1
High Income	50.1	28.7	46.7
**Insurance**^**a**^**(%)**			
Private	79.4	59.8	76.4
Public	7.5	17.2	9.0
Uninsured	13.1	23.1	14.6
**Physical Health**^**a**^**(%)**			
Excellent	28.8	29.7	29.0
Very Good	36.2	31.6	35.5
Good	25.0	27.4	25.4
Fair	7.5	9.2	7.8
Poor	2.4	2.1	2.4
**Mental Health**^**a**^**(%)**			
Excellent	40.0	44.0	40.7
Very Good	31.4	26.6	30.7
Good	22.2	23.0	22.3
Fair	5.1	5.1	5.1
Poor	1.2	1.4	1.3
**Region**^**a**^**(%)**			
Northeast	19.3	16.7	18.9
Midwest	26.4	16.9	24.9
South	34.2	56.6	37.7
West	20.1	9.9	18.5

^a^Differences between non-Latino Whites significant at *p* < 0.05 based on survey adjusted t-test for continuous (age) and chi-squared tests for the categorical variables

**Table 2:** Summary of estimated models testing differences in PCMH domains between Black and non-Latino White Men

	Model 1	Model 2	Model 3
**Personal primary care provider**			
Usual Source of Care (USC)	✓	✓	✓
Personal Provider	✓	✓	✓
Provider not a specialist	✓	✓	ns
**Enhanced Access to Care**			
Easy Phone Access	ns	ns	ns
Night/Weekend Hours	ns	ns	✓
Easy Contact After Regular Hours	✓	✓	ns
**Patient-Provider (Quality) Communication**			
Dr. Listens Carefully	✓	✓	ns
Dr. Explains Comprehend	✓	✓	ns
Dr. Shows Respect	✓	✓	ns
Dr. Spends Enough Time	✓	✓	✓
**Care is patient centered**			
Dr. Helps Treatment Decisions	✓	✓	✓
Dr. Asks Other Treatments	ns	ns	ns
Dr. Explains Options	ns	ns	ns
Dr. Respects Choices	ns	ns	ns
**Care is coordinated by USC**			
Go USC for New Health Problems	✓	✓	✓
Go USC for Preventive	✓	ns	ns
Go USC for Referral	✓	ns	ns
Go USC for Ongoing	ns	ns	ns
**Care is comprehensive (When needed)**			
Immediate Care Received	✓	✓	✓
Routine Care Received	✓	✓	✓
Tests Received	✓	✓	✓
Specialist Care Received	✓	✓	✓

Results are based on survey logistic regression models using non-Latino White and Black participants ages 18–64 years from the 2008–2016 Medical Expenditures Panel Survey. Model 1 is unadjusted. Model 2 adjusts for age. Model 3 is fully adjusted and includes all variables as specified in the covariates section

✓Indicates statistically significant difference (*p* < 0.05). “ns” indicates statistically not significant

Detailed information about survey questions, logical skips, and unweighted sample sizes are provided in Additional file 1: Figure S1.

**Table 3:** Summary of the relative and absolute differences in probabilities of PCMH care indicators, and the estimated explained proportion of absolute differences accounted for by predisposing, enabling, health need, and control factors

Domain	Measure	NLWs (Crude %)	Blacks (Crude %)	Absolute	Relative	Explained	Unexplained
				Crude	Crude		
				Difference	Difference		
				(%)	(%)		
Personal primary care provider	Usual Source of Care	72.5	60.4	12.1	16.7	60.9	39.1
Personal primary care provider	Personal Provider	48.4	40.7	7.7	15.9	8.7	91.3
Care is comprehensive	Immediate Care When Needed	87.6	81.9	5.7	6.5	35.3	64.7
Care is comprehensive	Specialist When Needed	85.2	80.0	5.2	6.1	34.5	65.5
Care is patient centered	Dr. Helps Treatment Decision	86.1	81.3	4.8	5.6	24.4	75.6
Care is comprehensive	Tests When Needed	92.6	88.4	4.3	4.6	40.1	59.9
Care is comprehensive	Routine Care When Needed	85.6	81.5	4.1	4.7	18.9	81.1
Enhanced Access to Care	Easy After Hours^a^	65.8	62.2	3.6	5.5	113.3	−13.3
Personal primary care provider	Provider Not a Specialist	90.9	87.8	3.0	3.4	47.9	52.1
Quality Communication	Dr. Spends Enough Time	90.0	87.3	2.7	3.0	46.8	53.2
Quality Communication	Dr. Explains Comprehend	94.4	91.9	2.5	2.6	69.5	30.5

Results are derived from Oaxaca decomposition techniques adapted for binary outcomes using Non-Latino Whites and Black participants ages 18–64 years from the 2008–2016 Medical Expenditures Panel Survey. For detailed information about survey questions for indicators and domains and logical skips leading to these differential Ns see detailed information provided in Additional file 1: Figure S1. Absolute difference is calculated based on the expected average crude probabilities as: 100*(Pr(non-Latino Whites) – Pr(Blacks)). The relative difference is calculated as (1-(Pr(Blacks)/Pr(non-Latino Whites))*100. Only measures with absolute difference ≥ 2% are presented in the Table

^a^An “Explained” % that exceeds 100 indicates that Blacks would have an advantage on that indicator after controlling for the factors included in the model

## Results

Descriptive characteristics are detailed in Table 1. Black males were on average younger than NLWs, less likely to report being married (35.7% vs 56.6%), and half as likely to report having a college education or more (15.9% vs 32.4%). Black males were also twice more likely to report being poor or near poor, half as likely to be classified as high-income, 2.3 times more likely to report being publicly insured and 1.8 times more likely to report being uninsured relative to NLW male counterparts. While differences in reported physical and mental health were statistically significantly different, differences were not substantively large.

### Group differences

Interesting differences in healthcare experiences were revealed in the current study (Table 2). The crude, age adjusted, and fully adjusted odds ratios and their 95% confidence intervals are presented in Additional file 1: Tables S1–S6. To facilitate visualization of our results, the crude and fully adjusted odds ratios and their 95% confidence intervals are presented in Fig. 1, and the estimated differences in marginal probabilities for each outcome between Black and NLW males and their 95 confidence intervals. The crude estimates revealed that Black and NLW males reported similar experiences on measures of enhanced access to care, care coordination, and, with the exception of differences in experiences related to decisions related to help on treatment options, on patient-centeredness of care. Black and NLW males differed on reports of access to personal primary care, quality patient-provider communication, and comprehensiveness of care. Substantial proportions of the reported differences were unexplained by the model covariates. The absolute and crude differences on the underlying measures with statistically significant differences are reported in Table 3.

### Explained differences in primary care access (Fig. 2)

Adjusting the models for the covariates explained 60.9, and 47.9% of the differences in, respectively, reported access to a usual provider of care, and the provider not being a specialist. Model covariates, however, explained a very small proportion (8.7%) of the difference in reporting a personal provider. The detailed decomposition indicated that the majority of the explained disparity in access to a usual provider was due to characteristic differences, between NLWs and Blacks, in predisposing (17.8%) and enabling (34.4%) factors. These factors also, respectively, explained 37.9 and 12.0% of the differences in reporting a non-specialist provider and the group differences on this indicator became statistically insignificant.

### Explained differences in provider quality communication

Adjusting the models for the covariates explained 46.8, and 69.5% of the differences in indicators of quality patient-provider communication; spends enough time with the patient, and explain issues in an easy to understand manner, respectively. For both measures the majority of explained differences stemmed from advantageous predisposing (11.9 and 21.8%, respectively) and enabling characteristics (23.2, and 27.8% respectively) among NLW males. Despite the above reported attenuation in differences after controlling for covariates the differences between NLW and Black males remained statistically significant.

### Explained differences in comprehensiveness of care

Finally, there were marked differences in timely access to comprehensive care. Adjusting the models for the covariates explained 35.3, 34.5, 40.1, and 18.9% of the differences in indicators of care comprehensiveness; immediate, specialist, tests or treatment, and routine care, respectively. For all these measures the majority of explained differences stemmed from advantageous enabling factors (30.5, 22.2, 26.2, and 9.8%, respectively) and, to a lesser extent, predisposing characteristics among NLW males. The differences between NLW and Black males remained statistically significant despite the above reported attenuations resulting from covariates adjustment.

## Discussion

In the current study, we examined racial differences in men’s experiences of patient-centered care broadly, and with defining features of the patient-centered medical home model in particular. Few studies have extracted a nuanced understanding of patient-centered experiences for Black men, particularly with regard to care coordination and provider interactions. Our analysis revealed distinct differences in health care access, patient-provider interactions, and comprehensiveness of care and care coordination between NLW and Black males. Overall, differences between groups were attenuated but not eliminated by accounting for the *Behavioral Model* factors. Notably, and reflecting the IOM definition of disparities, group health characteristics were not a primary driver of racial differences in care experiences across all these domains.

### Access to primary care providers

Black men were less likely than their NLW counterparts to have access to a usual or specific health care provider that was not a specialist. This is particularly concerning given that the vast majority of routine preventive health care and management of common chronic illnesses such as diabetes, hypertension, and mental health occur in a primary care context. Further, the Black men in this study were more likely to be of lower socioeconomic status (SES) as marked by less education, lower income, and being publicly insured. Recent studies have demonstrated that when primary care practices implement patient-centered medical home models, they are uniquely effective in raising preventive services utilization rates among low SES patients, including screening for colorectal cancer which disproportionately impacts Black men [27] . Black men’s utilization of preventive health services has been well studied but research on Black men’s general lack of connectedness to the primary care context is still insufficient. Specifically, having a relationship with a single consistent health care provider or team who can track health trajectories, symptoms, and manage care over time plays a significant and direct role in reducing disparities. This is also important because patients connected to a specific physician are more likely to receive care consistent with clinical guidelines [28]; and continuity of care is also strongly associated with fewer emergency department visits, inpatient hospitalizations, fewer complications from treatment, and lower health care costs for heart disease, chronic obstructive pulmonary disease, and diabetes [29].

### Quality of patient-provider communication

In this study, Black men who reported a usual source of care were less likely to perceive that their health provider spends enough time with them, and to explain issues in a way that they can understand. However, these differences were not very large. These findings are consistent with research showing that as a result of implicit bias, some physicians are less patient-centered in their communication style and content with their Black than with their White patients [8, 30] . Despite the small effects, the differences in communication quality combined with a higher perception among Black men that providers are less helpful in making treatment decisions have the potential to perpetuate historical mistrust, creating a synergistic dissuading effect on establishing a personal and continuous source of care and engaging in routine interactions with the health care system. High quality patient-provider communication empowers patients to more fully participate in their own care decision-making [31]. Empowering patients is also one of the central tenets of patient-centered care and the foundation upon which patient-centered medical homes models are built.

### Comprehensiveness of care

Finally, Black men in the current study experienced poorer outcomes than their NLW counterparts related to their access to both immediate and routine health care, access to medical tests and specialty care. Unmet subspecialty care needs contribute to the disparities experienced by medically underserved populations such as Black men. Generally, research on care comprehensiveness needs, barriers, adherence to care management plans and related decision-making by or on behalf of Black men is virtually nonexistent. Patient navigation interventions have shown particular promise for improving the coordination and completion of cancer screening for Black men specifically [32]. However, evidence is lacking on similar efforts to support the prevention or treatment of other chronic health conditions with high or rising prevalence in this population. Black men would undoubtedly benefit from greater support and advocacy while navigating the health care system. Specifically, systemic care coordination efforts should take into consideration unique needs related to age, health literacy, socioeconomic status, insurance gaps, and other layers that account for the impact of environmental context and identity on health outcomes.

### Limitations

We point to three major limitations to this work. First, all measures used in our analyses are based on self-reported survey data. Self-reports are subject to recall and other biases which could have affected our estimates. Sensitivity studies by AHRQ indicate good response validity for MEPS data [33]. Second, our measures do not cover the entire spectrum of the operational definitions of a patient-centered care or the medical home and its domains. We used indicators as determined and published in previous work on patient centered care in MEPS. More expansive definitions, using other data sources, can potentially uncover patterns and differences not attended to in this work. Finally, we used a modified version of the Behavioral Model given available data in the MEPS. As such, several measures (e.g. contextual and health system level factors) were omitted, which explains the high levels of unexplained differences reported in our study. Any inferences about the contribution of these critical factors are omitted from our discussion and would require further research.

## Conclusions

Consensus is growing that patient-centered medical home models of delivering care are on balance, positive for patient outcomes and potentially reduce health disparities. They have been increasingly integrated across healthcare settings as the “gold-standard” of care. Our findings show that Black men, particularly those who are socioeconomically disadvantaged, are not equitably experiencing the core standards of this model and point to specific areas where Black men are not being adequately served by the health care system. Our findings suggest that health care team members, including community advocates, may need to better support Black men in connecting to a primary care physician, and once connected, in coordinating their comprehensive care and communicating their questions and concerns to optimize their medical care and patient experience.

## Supplementary information

**Additional file 1: Figure S1.** Detailed list of the questions, and the original scaling, used to assess each of the six domains of health care experiences. **Table S1.** Logistic Regression Models for personal primary care provider domain indicators using Non-Latino Whites and Black participants ages 18–64 years from the 2008–2016 Medical Expenditures Panel Survey. **Table S2.** Logistic Regression Models for enhanced access to care domain indicators using Non-Latino Whites and Black participants ages 18–64 years from the 2008–2016 Medical Expenditures Panel Survey. **Table S3.** Logistic Regression Models for patient-provider communication domain indicators using Non-Latino Whites and Black participants ages 18–64 years from the 2008–2016 Medical Expenditures Panel Survey. **Table S4.** Logistic Regression Models for patient centered care domain indicators using Non-Latino Whites and Black participants ages 18–64 years from the 2008–2016 Medical Expenditures Panel Survey. **Table S5.** Logistic Regression Models for patient care coordination indicators using Non-Latino Whites and Black participants ages 18–64 years from the 2008–2016 Medical Expenditures Panel Survey. **Table S6.** Logistic Regression Models for care comprehensiveness indicators using Non-Latino Whites and Black participants ages 18–64 years from the 2008–2016. **Table S7.** Detailed Results from Oaxaca decomposition techniques adapted for binary outcomes using Non-Latino Whites and Black participants ages 18–64 years from the 2008–2016 Medical Expenditures Panel Survey. Medical Expenditures Panel Survey.

## Data Availability

All data generated and analyzed during the current study, and their codebooks and documentation, are publicly available from the Agency for Healthcare Research and Quality (AHRQ).

## Abbreviations

AHRQ: Agency for Healthcare Research and Quality
MEPS: Medical Expenditure Panel Survey
NLW: Non-Latino White
PCMH: Patient Centered Medical Home
PCC: Patient Centered Care

## References

[1] Berwick DM (2002). A user’s manual for the IOM’s ‘quality chasm’ report. Health Affairs (Project Hope).

[2] Starfield B (2011). Is patient-centered care the same as person-focused care?. Perm J.

[3] McMillan SS, Kendall E, Sav A, King MA, Whitty JA, Kelly F (2013). Patient-centered approaches to health care: a systematic review of randomized controlled trials. Med Care Res Rev.

[4] Morgan S, Yoder LH (2012). A concept analysis of person-centered care. J Holist Nurs.

[5] Saha S, Beach MC, Cooper LA (2008). Patient centeredness, cultural competence and healthcare quality. J Natl Med Assoc.

[6] Johnson RL, Roter D, Powe NR, Cooper LA (2004). Patient race/ethnicity and quality of patient–physician communication during medical visits. Am J Public Health.

[7] Martin KD, Roter DL, Beach MC, Carson KA, Cooper LA (2013). Physician communication behaviors and trust among black and white patients with hypertension. Med Care.

[8] Cooper LA, Roter DL, Carson KA, Beach MC, Sabin JA, Greenwald AG (2012). The associations of clinicians’ implicit attitudes about race with medical visit communication and patient ratings of interpersonal care. Am J Public Health.

[9] Fowler-Brown A, Ashkin E, Corbie-Smith G, Thaker S, Pathman DE (2006). Perception of racial barriers to health care in the rural south. J Health Care Poor Underserved.

[10] Musa D, Schulz R, Harris R, Silverman M, Thomas SB (2009). Trust in the Health Care System and the use of preventive health services by older black and White adults. Am J Public Health.

[11] Cuffee YL, Hargraves JL, Rosal M, Briesacher BA, Schoenthaler A, Person S (2013). Reported racial discrimination, Trust in Physicians, and medication adherence among Inner-City African Americans with hypertension. Am J Public Health.

[12] Martins D, Agodoa L, Norris K (2012). Chronic kidney disease in disadvantaged populations. Int J Nephrol.

[13] Kochanek KD, Arias E, & Anderson RN. Leading causes of death contributing to decrease in life expectancy gap between black and White populations: United States, 1999–2013: US Department of Health and Human Services, centers for disease control and Prevention, National Center for Health Statistics; 2015. https://www.cdc.gov/nchs/data/databriefs/db218.pdf.

[14] Hammond WP, Mohottige D, Chantala K, Hastings JF, Neighbors HW, Snowden L (2011). Determinants of usual source of care disparities among African American and Caribbean black men: findings from the National Survey of American life. J Health Care Poor Underserved.

[15] Cheatham CT, Barksdale DJ, Rodgers SG (2008). Barriers to health care and health-seeking behaviors faced by black men. J Am Acad Nurse Pract.

[16] Stange KC, Nutting PA, Miller WL, Jaén CR, Crabtree BF, Flocke SA (2010). Defining and measuring the patient-centered medical home. J Gen Intern Med.

[17] Jackson GL, Powers BJ, Chatterjee R, Bettger JP, Kemper AR, Hasselblad V (2013). The patient-centered medical home: a systematic review. Ann Intern Med.

[18] Scholle SH, Torda P, Peikes D, Han E, Genevro J (2010). Engaging patients and families in the medical home.

[19] Tarraf W, Jensen G, González HM (2017). Patient centered medical home care among near-old and older race/ethnic minorities in the US: findings from the medical expenditures panel survey. J Immigr Minor Health.

[20] Jones AL, Cochran SD, Leibowitz A, Wells KB, Kominski G, Mays VM (2015). Usual primary care provider characteristics of a patient-centered medical home and mental health service use. J Gen Intern Med.

[21] Romaire MA, Bell JF (2010). The medical home, preventive care screenings, and counseling for children: evidence from the medical expenditure panel survey. Acad Pediatr.

[22] Romaire MA, Bell JF, Grossman DC (2012). Medical home access and health care use and expenditures among children with special health care needs. Arch Pediatr Adolesc Med.

[23] Beal A, Hernandez S, Doty M (2009). Latino access to the patient-centered medical home. J Gen Intern Med.

[24] Andersen R, Newman JF (2005). Societal and individual determinants of medical care utilization in the United States. Milbank Q.

[25] Babitsch B, Gohl D, von Lengerke T (2012). Re-revisiting Andersen’s behavioral model of health services use: a systematic review of studies from 1998–2011. GMS Psycho-Soc Med.

[26] Jann B (2008). The blinder–Oaxaca decomposition for linear regression models. Stata J.

[27] Markovitz AR, Alexander JA, Lantz PM, Paustian ML (2015). Patient-centered medical home implementation and use of preventive services: the role of practice socioeconomic context. JAMA Intern Med.

[28] Atlas SJ, Grant RW, Ferris TG, Chang Y, Barry MJ (2009). Patient–physician connectedness and quality of primary care. Ann Intern Med.

[29] Hussey PS, Schneider EC, Rudin RS, Fox DS, Lai J, Pollack CE (2014). Continuity and the costs of care for chronic disease. JAMA Intern Med.

[30] Chapman EN, Kaatz A, Carnes M (2013). Physicians and implicit bias: how doctors may unwittingly perpetuate health care disparities. J Gen Intern Med.

[31] Street RL, Makoul G, Arora NK, Epstein RM (2009). How does communication heal? Pathways linking clinician–patient communication to health outcomes. Patient Educ Couns.

[32] Cole H, Thompson HS, White M, Browne R, Trinh-Shevrin C, Braithwaite S (2017). Community-based, preclinical patient navigation for colorectal cancer screening among older black men recruited from barbershops: the MISTER B trial. Am J Public Health.

[33] Zuvekas SH, Olin GL (2009). Validating household reports of health care use in the medical expenditure panel survey. Health Serv Res.

