# Supplementary material for: Identifying disparities in patient-centered care experiences between non-Latino white and black men: results from the 2008-2016 Medical Expenditure Panel Survey
Source: BMC Health Serv Res. 2020 Jun 3;20:495. doi: 10.1186/s12913-020-05357-5 (PMC7268709; doi:10.1186/s12913-020-05357-5)
Supplement: Supplementary file 1 — Additional file 1: Figure S1. Detailed list of the questions, and the original scaling, used to assess each of the six domains of health care experiences. Table S1. Logistic Regression Models for personal primary care provider domain indicators using Non-Latino Whites and Black participants ages 18–64 years from the 2008–2016 Medical Expenditures Panel Survey. Table S2. Logistic Regression Models for enhanced access to care domain indicators using Non-Latino Whites and Black participants ages 18–64 years from the 2008–2016 Medical Expenditures Panel Survey. Table S3. Logistic Regression Models for patient-provider communication domain indicators using Non-Latino Whites and Black participants ages 18–64 years from the 2008–2016 Medical Expenditures Panel Survey. Table S4. Logistic Regression Models for patient centered care domain indicators using Non-Latino Whites and Black participants ages 18–64 years from the 2008–2016 Medical Expenditures Panel Survey. Table S5. Logistic Regression Models for patient care coordination indicators using Non-Latino Whites and Black participants ages 18–64 years from the 2008–2016 Medical Expenditures Panel Survey. Table S6. Logistic Regression Models for care comprehensiveness indicators using Non-Latino Whites and Black participants ages 18–64 years from the 2008–2016. Table S7. Detailed Results from Oaxaca decomposition techniques adapted for binary outcomes using Non-Latino Whites and Black participants ages 18–64 years from the 2008–2016 Medical Expenditures Panel Survey. Medical Expenditures Panel Survey. [file 12913_2020_5357_MOESM1_ESM.zip › Legend Supplementary TablesR1.docx]

**Supporting Information**

**S1 Fig. Detailed list of the questions, and the original scaling, used to assess each of the six domains of health care experiences.**

**S1 Table.** **Logistic Regression Models for personal primary care provider domain indicators using Non-Latino Whites and Black participants ages 18-64 years from the 2008-2016 Medical Expenditures Panel Survey.**

**S2 Table. Logistic Regression Models for enhanced access to care domain indicators using Non-Latino Whites and Black participants ages 18-64 years from the 2008-2016 Medical Expenditures Panel Survey.**

**S3 Table. Logistic Regression Models for patient-provider communication domain indicators using Non-Latino Whites and Black participants ages 18-64 years from the 2008-2016 Medical Expenditures Panel Survey.**

**S4 Table. Logistic Regression Models for patient centered care domain indicators using Non-Latino Whites and Black participants ages 18-64 years from the 2008-2016 Medical Expenditures Panel Survey.**

**S5 Table. Logistic Regression Models for patient care coordination indicators using Non-Latino Whites and Black participants ages 18-64 years from the 2008-2016 Medical Expenditures Panel Survey.**

**S6 Table. Logistic Regression Models for care comprehensiveness indicators using Non-Latino Whites and Black participants ages 18-64 years from the 2008-2016**

**S7 Table. Detailed Results from Oaxaca decomposition techniques adapted for binary outcomes using Non-Latino Whites and Black participants ages 18-64 years from the 2008-2016 Medical Expenditures Panel Survey. Medical Expenditures Panel Survey.**
