# Supplementary material for: Identifying disparities in patient-centered care experiences between non-Latino white and black men: results from the 2008-2016 Medical Expenditure Panel Survey
Source: BMC Health Serv Res. 2020 Jun 3;20:495. doi: 10.1186/s12913-020-05357-5 (PMC7268709; doi:10.1186/s12913-020-05357-5)
Supplement: Supplementary file 1 — Additional file 1: Figure S1. Detailed list of the questions, and the original scaling, used to assess each of the six domains of health care experiences. Table S1. Logistic Regression Models for personal primary care provider domain indicators using Non-Latino Whites and Black participants ages 18–64 years from the 2008–2016 Medical Expenditures Panel Survey. Table S2. Logistic Regression Models for enhanced access to care domain indicators using Non-Latino Whites and Black participants ages 18–64 years from the 2008–2016 Medical Expenditures Panel Survey. Table S3. Logistic Regression Models for patient-provider communication domain indicators using Non-Latino Whites and Black participants ages 18–64 years from the 2008–2016 Medical Expenditures Panel Survey. Table S4. Logistic Regression Models for patient centered care domain indicators using Non-Latino Whites and Black participants ages 18–64 years from the 2008–2016 Medical Expenditures Panel Survey. Table S5. Logistic Regression Models for patient care coordination indicators using Non-Latino Whites and Black participants ages 18–64 years from the 2008–2016 Medical Expenditures Panel Survey. Table S6. Logistic Regression Models for care comprehensiveness indicators using Non-Latino Whites and Black participants ages 18–64 years from the 2008–2016. Table S7. Detailed Results from Oaxaca decomposition techniques adapted for binary outcomes using Non-Latino Whites and Black participants ages 18–64 years from the 2008–2016 Medical Expenditures Panel Survey. Medical Expenditures Panel Survey. [file 12913_2020_5357_MOESM1_ESM.zip › PCMH Supplemental Figure 1R1.docx]

Supplemental Figure 1. Details of survey questions and logical skips of measured PCMH domains.

**Personal Primary Care Provider**

- Type of Provider (N=31,038)
- [If Personal Provider] What is {PROVIDER}’s specialty? (N=14,149)

**Enhanced Access to Care**

- How difficult is it to contact {a medical person at} {PROVIDER} during regular business hours over the telephone about a health problem? (N=29,610)
- Does {PROVIDER} have office hours at night or on weekends? (N=27,124)
- How difficult is it to contact {a medical person at} {PROVIDER} after their regular hours in case of urgent medical needs? (N=20,375)

**Care is Coordinated: Situations Respondent Goes to USC**

- Is {PROVIDER} the {person/place} (READ NAME(S) ABOVE) would go to for New health problems? (N=30,992)
- Is {PROVIDER} the {person/place} (READ NAME(S) ABOVE) would go to for Preventive health care, such as general checkups, examinations, and immunizations? (N=30,983)
- Is {PROVIDER} the {person/place} (READ NAME(S) ABOVE) would go to for Referrals to other health professionals when needed? (N=30,963)
- Is {PROVIDER} the {person/place} (READ NAME(S) ABOVE) would go to for Ongoing health problems? (N=30,962)

(N=45,612)

Have a USC?

If **YES**

**Care is Patient Centered**

- If there were a choice between treatments, how often would {a medical person at} {PROVIDER} ask (READ NAME(S) BELOW) to help make the decision? (N=28,432)
- Does {someone at} {PROVIDER} usually ask about prescription medications and treatments other doctors may give them? (N=29,385)
- Does {a medical person at} {PROVIDER} present and explain all options to (READ NAME(S) BELOW)? (N=29,912)
- Thinking about the types of medical, traditional and alternative treatments that (READ NAME(S) BELOW) (is/are) are happy with, how often does a medical person at} {PROVIDER} show respect for these treatments? (N=26,952)

**Provider Quality Communication**

- In the last 12 months, how often did doctors or other health providers listen carefully to you? (N=20,881)
- In the last 12 months, how often did doctors or other health providers explain things in a way that was easy to understand? (N=20,940)
- In the last 12 months, how often did doctors or other health providers show respect for what you had to say? (N=20,936)
- In the last 12 months, how often did doctors or other health providers spend enough time with you? (N=20,911)

**If 1+** In the last 12 months, not counting the times you went to an emergency room, how many times did you go to a doctor’s office or clinic to get health care for yourself?

**If YES** In the last 12 months, not counting the times you needed care right away, did you make any appointments for your health care at a doctor’s office or clinic?

- In the last 12 months, when you needed care right away how often did you get care as soon as you thought you needed? (N=7,954)
- In the last 12 months, not counting the times you needed care right away, how often did you get an appointment for your health care at a doctor’s office or clinic as soon as you thought you needed? (N=18,635)
- In the last 12 months, how often was it easy to see a specialist that you needed to see? (N=10,003)

**If YES** Specialists are doctors like surgeons, heart doctors, allergy doctors, skin doctors, and others who specialize in one area of health care. In the last 12 months, did you or a doctor think you needed to see a specialist?

**If YES** In the last 12 months, did you have an illness, injury, or condition that needed care right away in a clinic, emergency room, or doctor’s office?

- In the last 12 months, how often was it easy to get the care, tests, or treatment you or a doctor believed necessary? (N=14,819)

**& if YES** In the last 12 months, did you or a doctor believe you needed any care, tests, or treatment?

**Care is Comprehensive**
